# Supplementary material for: NET Formation Was Reduced via Exposure to Extremely Low-Frequency Pulsed Electromagnetic Fields
Source: Int J Mol Sci. 2023 Sep 27;24(19):14629. doi: 10.3390/ijms241914629 (PMC10572227; doi:10.3390/ijms241914629)
Supplement: Supplementary file 1 [file ijms-24-14629-s001.zip › ijms-2614220-supplementary.pdf]

Supplementary Figure S1:

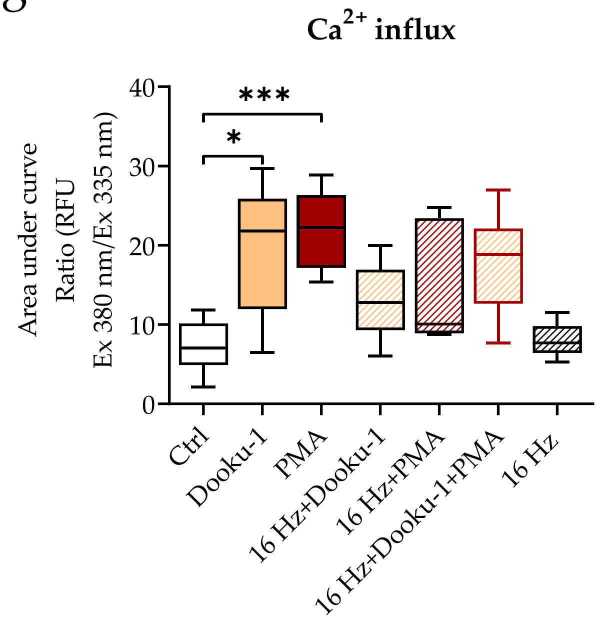

Supplementary Figure S1. Ca^{2+}-influx after incubation with Piezo1 inhibitor. Extended Figure to Figure 4D in the main manuscript with all controls shown. Ca^{2+}-influx was quantified by Fura-2-AM measurement over a time course from 10-70 min. Dooku-1: 20 µM, PMA: 100 nM, 16 Hz: 16 Hz ELF-PEMF exposure for 7 min. * $p < 0.05$, *** $p < 0.001$ as determined by non-parametric Kruskal-Wallis test.

Supplementary Figure S2:

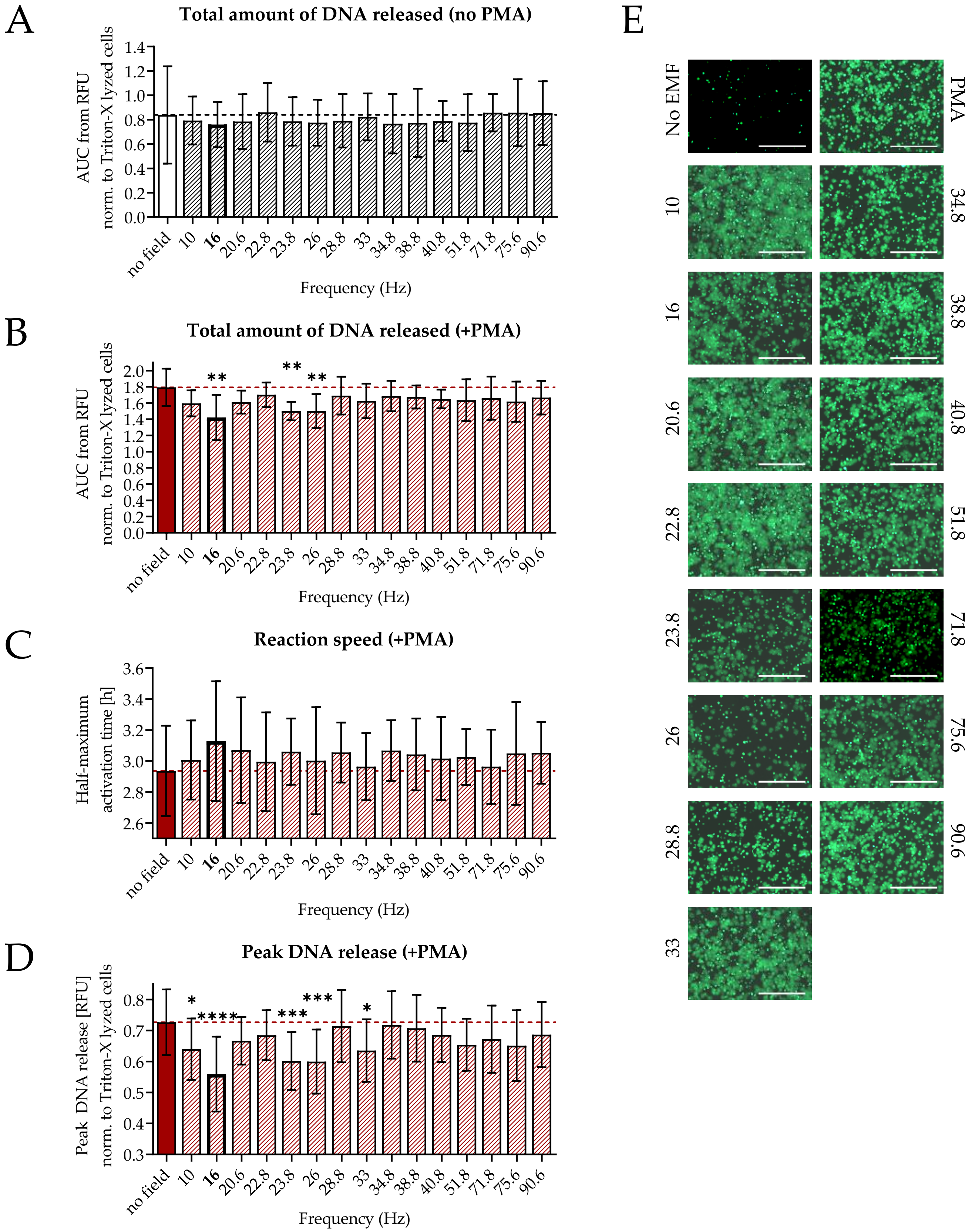

Supplementary Figure S2. ELF-PEMF Screening. **(A)** Total DNA release of non-treated cells obtained by Sytox Green assay. Cells were exposed to indicated frequencies for 7 min. N=11, n=3 **(B)-(D)** Different analysis parameters calculated from Sytox Green Assay with addition of 100 nM PMA ± 7 min of 16 Hz ELF-PEMF exposure. N=10, n=3 **(B)** Total amount of released DNA **(C)** Reaction speed **(D)** Peak DNA release **(E)** Exemplary images from Sytox Green assay at 3 h time point, green fluorescence indicates DNA release. Scale bar: 200 µm. * $p < 0.05$, ** $p < 0.01$, *** $p < 0.001$ and ****$p < 0.0001$ as determined by Friedman test.

Supplementary Figure S3:

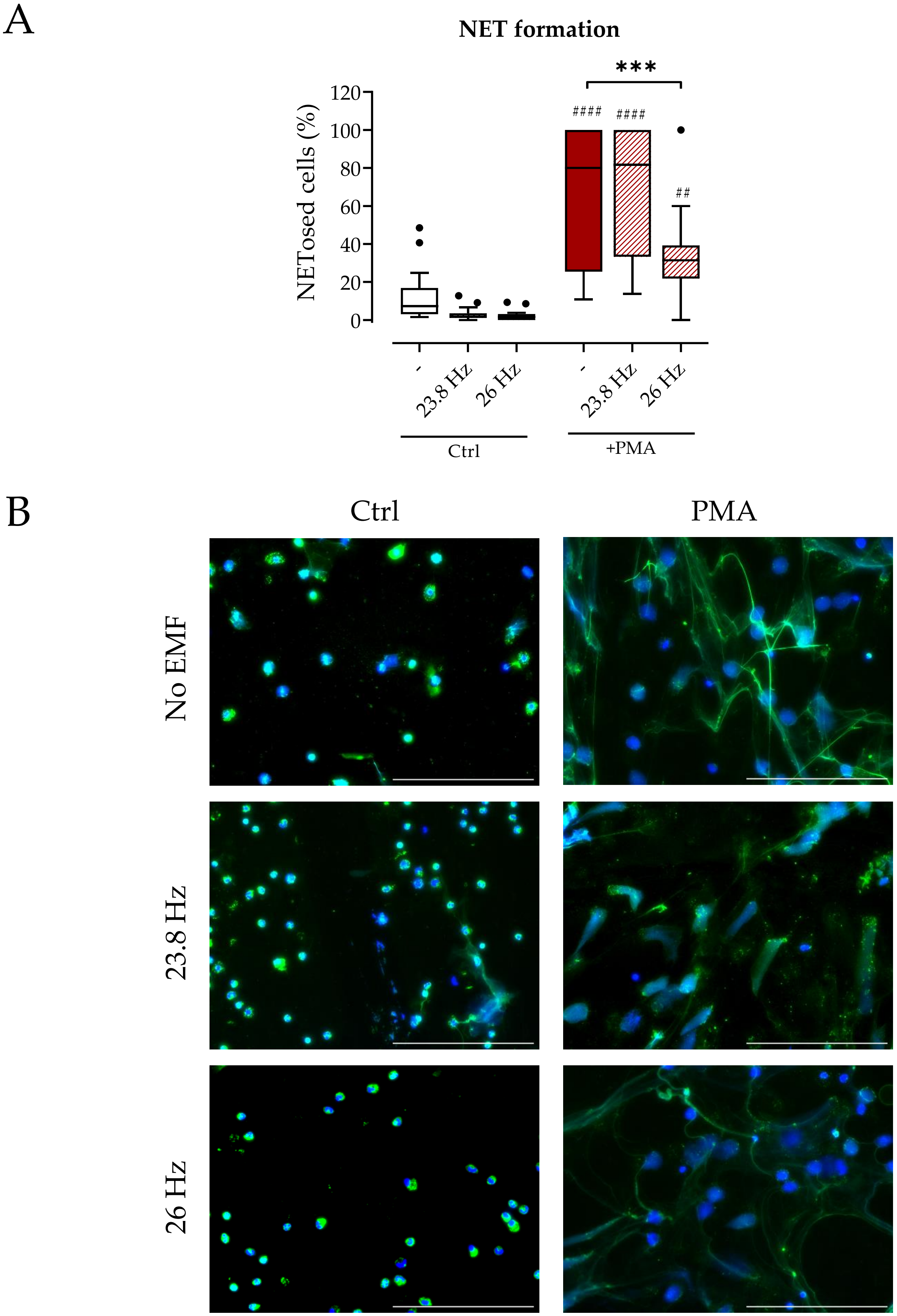

Supplementary Figure S3. Detailed analysis shows no effect of two other ELF-PEMF on NET formation. **(A)** NET formation was obtained from immunofluorescence images, ± 100 nM PMA and ± 7 min of exposure of ELF-PEMF in indicated frequencies. N=3, n=3. **(B)** Exemplary images of immunofluorescence staining, ± 100 nM PMA and ± ELF-PEMF exposure. N=3, n=3. Blue: DNA (Hoechst 33342), green: Myeloperoxidase. Scale bar: 200 µm. Statistical analysis was done using Two Way ANOVA. *** $p < 0.001$, ## $p<0.01$ and #### $p<0.0001$ to respective control exposed to the same ELF-PEMF without PMA.
